# Angiotensin-(1-7) Inhibits Thrombin-Induced Endothelial Phenotypic Changes and Reactive Oxygen Species Production via NADPH Oxidase 5 Downregulation

**DOI:** 10.3389/fphys.2017.00994

**Published:** 2017-12-08

**Authors:** Wan-Yu Pai, Wan-Yu Lo, Todd Hsu, Ching-Tien Peng, Huang-Joe Wang

**Affiliations:** ^1^Department of Bioscience and Biotechnology, Center of Excellence for the Oceans, National Taiwan Ocean University, Keelung, Taiwan; ^2^Cardiovascular and Translational Medicine Laboratory, Department of Biotechnology, Hungkuang University, Taichung, Taiwan; ^3^Department of Pediatrics, Children's Hospital, China Medical University and Hospital, Taichung, Taiwan; ^4^Department of Biotechnology, Asia University, Taichung, Taiwan; ^5^Department of Internal Medicine, School of Medicine, China Medical University, Taichung, Taiwan; ^6^Division of Cardiovascular Medicine, Department of Internal Medicine, China Medical University and Hospital, Taichung, Taiwan; ^7^Cardiovascular Research Laboratory, China Medical University and Hospital, Taichung, Taiwan

**Keywords:** human aortic endothelial cells, thrombin, angiotensin-(1-7), reactive oxygen species, NADPH oxidase 5

## Abstract

**Background and Aims:** The angiotensin-(1-7)/angiotensin-converting enzyme 2/Mas receptor axis counter-regulates the detrimental effects of angiotensin II. Beneficial effects of angiotensin-(1-7), including anti-inflammation, oxidative stress reduction, and anti-thrombosis, have been reported. Previous studies documented that ramipril decreased thrombin generation in human hypertension and that the anti-thrombotic effects of captopril and losartan were angiotensin-(1-7)-dependent, suggesting an interaction between thrombin and angiotensin-(1-7). However, it is not clear whether angiotensin-(1-7) can alleviate the endothelial phenotypic changes induced by thrombin. We have previously documented cytoskeleton remodeling, cell adhesion, and cell migration as dominant altered phenotypes in thrombin-stimulated human aortic endothelial cells (HAECs). In this study, we investigated whether angiotensin-(1-7) can modulate thrombin-induced phenotypic changes. Furthermore, we investigated whether NAPDH oxidase 5 (Nox5)-produced reactive oxygen species (ROS) play a significant role in angiotensin-(1-7)-mediated phenotypic changes.

**Methods:** HAECs were pretreated with 100 nM angiotensin-(1-7) for 1 h, followed by stimulation with 2 units/mL thrombin for different times. Immunofluorescent assay, monocyte adhesion assay, wound-healing assay, ROS assay, real-time PCR, Western blotting, and Nox5 siRNA transfection were conducted. HAECs were pretreated with the ROS scavenger N-acetylcysteine (NAC) to determine whether thrombin-induced phenotypic changes depended on ROS production.

**Results:** Angiotensin-(1-7) prevented thrombin-induced actin cytoskeleton derangements, monocyte adhesion, and migratory impairment. Nox5 siRNA transfection confirmed that thrombin-induced Nox5 expression stimulated ROS production and increased HO-1/NQO-1/ICAM-1/VCAM-1 gene expression, all of which were decreased by angiotensin-(1-7). Phenotypic changes induced by thrombin were prevented by NAC pretreatment.

**Conclusion:** Angiotensin-(1-7) prevents actin cytoskeleton derangement, monocyte adhesion, and migration impairment induced by thrombin via downregulation of ROS production. In addition, thrombin-induced Nox5 expression is involved in the production of ROS, and angiotensin-(1-7) decreases ROS through its inhibitory effect on Nox5 expression.

## Introduction

Atherosclerosis is a chronic inflammatory disease initiated through endothelial dysfunction caused by several factors, including low-density lipoproteins, hyperglycemia, oscillatory shear stress, oxidants, tumor necrosis factor-α, endotoxin, and thrombin (Steffel et al., 2006; Hess and Grant, 2011). In later stages, atherosclerotic plaques undergo spontaneous rupture with inflammatory substances such as tissue factor contacting with the circulating clotting factor VIIa. The tissue factor:VIIa complex may activate coagulation factors IX and Xa. Factor Xa further catalyzes the production of thrombin, resulting in the formation of fibrin (Steffel et al., 2006). This coagulation chain reaction results in thrombus formation and evolves into atherothrombosis that presents as acute coronary syndrome, acute ischemic stroke, and acute limb ischemia (Viles-Gonzalez et al., 2004). Under clinical conditions, ruptured plaques with intraplaque hemorrhage can rapidly increase in size and induce an inflammatory response through an auto-amplifying cascade, wherein thrombin plays a significant role (Hayden and Tyagi, 2004).

Thrombin plays a major role in coagulation and causes multiple endothelial phenotypic changes, including cell contraction, enhanced permeability, endothelial-dependent relaxation, leukocyte adhesion, migration, proliferation, and angiogenesis, suggestive of its key role in atherogenesis (Minami et al., 2004; Borissoff et al., 2009). Production of reactive oxygen species (ROS) from multiple insults may cause endothelial dysfunction and promote atherogenesis (Harrison et al., 2003; Sena et al., 2013). Most ROS produced in the vascular system originate from the mitochondrial electron transport chain, xanthine oxidase, and nicotinamide adenine dinucleotide phosphate (NADPH) oxidases (Noxs). Nox-derived ROS, in particular the superoxide anion and hydrogen peroxide, are well-known signaling molecules that modulate endothelial phenotypic changes (Dworakowski et al., 2008). Endothelial exposure of thrombin has been reported to produce substantial elevation in the release of Nox-dependent superoxide anion and hydrogen peroxide (Holland et al., 1998). Among the seven known Nox subtypes, Nox1, Nox2, Nox4, and Nox5 are expressed in endothelial cells and are important ROS sources in the cardiovascular system (Bedard and Krause, 2007). Recently, BelAiba et al. reported that both Nox2 and Nox5 are equally important in the generation of endothelial ROS in thrombin-stimulated human microvascular endothelial cells (BelAiba et al., 2007).

The renin-angiotensin-aldosterone system (RAAS) is a major component in human physiological and pathological responses of the cardiovascular system and is implicated in endothelial dysfunction, atherosclerosis, hypertension, and congestive heart failure. Angiotensin II is a major effector of RAAS and acts mainly via angiotensin II type I receptor (AT1R) to mediate pro-inflammatory and pro-thrombotic effects, and to increase oxidative stress (Mehta and Griendling, 2007). The angiotensin-(1-7)/angiotensin-converting enzyme 2 (ACE2)/Mas receptor axis is a recently discovered pathway that counter-regulates the detrimental effects of angiotensin II (Rabelo et al., 2011). In this pathway, angiotensin-(1-7) is produced from angiotensin II or angiotensin I through the catalytic activity of ACE2, and binding of angiotensin-(1-7) to the Mas receptor mediates its multiple beneficial effects (Santos et al., 2003), including anti-inflammatory effects (Al-Maghrebi et al., 2009; El-Hashim et al., 2012; Fraga-Silva et al., 2014), reduction of oxidative stress (Benter et al., 2008; Meng et al., 2015; Zhang Y. et al., 2015), and anti-thrombotic effects (Kucharewicz et al., 2002; Fraga-Silva et al., 2008; Fang et al., 2013). Recent studies have also revealed that angiotensin-(1-7) can inhibit atherosclerotic lesion progression and increase atherosclerotic plaque stability (Yang et al., 2013; Zhang F. et al., 2015).

Senchenkova et al. documented that chronic angiotensin II infusion enhanced thrombus generation in mouse microvasculature (Senchenkova et al., 2010). Ekholam et al. reported that long-term ACE inhibition with ramipril decreased thrombin generation in human hypertension (Ekholm et al., 2002). In addition, Kucharewicz et al. found that the anti-thrombotic effects of the ACE inhibitor captopril and the angiotensin receptor blocker losartan were dependent on the effects of angiotensin-(1-7) (Kucharewicz et al., 2002). Together, these studies suggested an interaction between thrombin and angiotensin-(1-7). However, it is not clear whether the beneficial effects of angiotensin-(1-7) play a role in modulating acute cellular stresses in thrombin-stimulated human aortic endothelial cells (HAECs).

Isobaric tags for relative and absolute quantification (iTRAQ) is a gel-free approach for high-throughput quantitative proteomics analysis (Aggarwal et al., 2006). We have previously documented the feasibility of the iTRAQ technique coupled with MetaCore™ pathway analysis software for evaluating acute endothelial responses to thrombin stimulation. In that study, we identified that cytoskeleton remodeling, cell adhesion, and cell migration are the dominant altered phenotypes in thrombin-stimulated HAECs (Wang et al., 2015). In this study, we aimed to investigate the role of angiotensin-(1-7) in mediating the modulation of these altered phenotypes in thrombin-stimulated HAECs. In addition, we examined the underlying mechanisms to determine whether Nox5-produced ROS play an important role in mediating effects of angiotensin-(1-7) on thrombin-induced phenotypic changes.

## Materials and methods

### Endothelial cell culture and sample treatment

Human aortic endothelial cells (HAECs) were purchased from Cell Applications, Inc., (San Diego, CA, USA) and were cultured in endothelial cell growth medium (Cell Applications, Inc.) according to the manufacturer's recommendations. All chemicals were obtained from Sigma-Aldrich (St. Louis, MO) unless otherwise specified. HAECs were stimulated with human alpha-thrombin at a dose of 2 U/mL for different times with or without 100 nM angiotensin-(1-7) pretreatment for 1 h.

### Visualization of actin cytoskeleton remodeling by confocal microscopy

The actin cytoskeleton was visualized by staining the cells with AlexaFluor®488 phalloidin (A12379; Thermo Fisher/Invitrogen). HAECs were seeded on coverslips at a density of 5 × 10^4^ cells/mm, followed by stimulation with thrombin (2 U/mL) for 5 h with or without 100 nM angiotensin-(1-7) pretreatment 1 h before the thrombin stimulation. After the treatments, the HAECs were rinsed in phosphate-buffered saline (PBS) and fixed for 10 min in 3.7% paraformaldehyde in PBS. After two rinses, the HAECs were permeabilized in 0.1% Triton X-100 in PBS for 10 min. The HAECs were incubated for 20 min at 37°C with AlexaFluor®488-phalloidin (15 U/mL) in PBS, followed by three washes in PBS. For the last wash, PBS was supplemented with propidium iodide (4 μg/mL) to stain the nuclei. The morphology of treated cells was studied by confocal microscopy using a Leica TCS SP8 confocal laser-scanning microscope with an HC PL APO 40×/1.30 Oil CS2 objective lens (Leica Microsystems, Germany). For excitation of AlexaFluor®488, we utilized the Leica supercontinuum white light laser visible excitation laser line (488 nm) at an intensity level of 3%. Emission signal was captured with a Leica HyD detector with a set emission wavelength gap (505–555 nm). All images were acquired using Leica Application Suite X (version 2.0.1.14392, LAS X).

### Monocyte adhesion assay

Adhesion experiments were performed with monocytic THP-1 cells using calcein acetoxymethyl ester (Calcein-AM; Molecular Probes, Eugene, OR, USA) labeling as previously described (Wang et al., 2014). Briefly, THP-1 cells were stained with the dye at a concentration of 7.5 μM for 30 min immediately preceding the adhesion assay. HAECs were maintained in 12-well plates until confluence. The HAECs (10^5^ cells/well) were then stimulated with thrombin (2 U/mL) for 5 h with or without 100 nM angiotensin-(1-7) pretreatment 1 h before the thrombin stimulation and incubated with culture medium containing the labeled THP-1 cells (THP-1/HAECs = 7) for 10 min. Non-adherent THP-1 cells were removed by washing with PBS for 20 s. Adherent THP-1 cells were visualized and quantified in 10 randomly viewed fields by fluorescence microscopy (OLYMPUS, Japan).

### Wound-healing migration assay

Migration assay was performed by incubating HAECs at 1 × 10^5^ cells/well in 12-well plates for 24 h before stimulation. After treatment, a “wound” was created in confluent cells by scratching each well with a spatula (1.4 mm), followed by incubation with endothelial cell growth medium. Cells were photographed at different times using an OLYMPUS DP72 microscope. The migratory ability of cells was evaluated by measuring the width of the wounds at 0, 6, 24, and 48 h. Data were analyzed using ImageJ (National Institute of Health, Bethesda, MD, USA) and are expressed as a percentage of migratory distance of the corresponding treatment at 0 h, which was set as 100%.

### Measurement of ROS induction

Human aortic endothelial cells (HAECs) were plated at 1 × 10^5^ cells/well in 12-well plates and incubated for 24 h before treatments. The cells were stimulated with thrombin (2 U/mL) for 5 h with or without 100 nM angiotensin-(1-7) pretreatment 1 h before the thrombin stimulation. The cells were loaded with the redox-sensitive probe 2′,7′-dichlorodihydronfluorescein diacetate (DCFH-DA) to measure intracellular ROS. After the treatments, all groups were incubated with 10 μM DCFH-DA at 37°C for 30 min in dark. Unbound DCFH-DA was removed by two washes with PBS. DCFH-DA fluorescence was visualized with a fluorescence microscope (OLYMPUS DP72) equipped with a digital camera. Fluorescence intensity (ROS activity) was quantified with ImageJ and expressed as fold changes of the corresponding control.

### Real-time polymerase chain reaction (PCR)

The mRNA expression levels of genes of interest in the HAECs were analyzed by real-time PCR. All PCRs were performed using the StepOnePlus Real-Time PCR instrument (Applied Biosystems, CA). PCR conditions were defined according to parameters recommended by the manufacturer. Gene expression levels were comparatively analyzed using StepOne software v2.2. The glyceraldehyde-3-phosphate dehydrogenase house-keeping gene (*GAPDH*) was selected as the internal control. Target gene expression, normalized to the endogenous internal control and relative to a reference sample, was auto-calculated using the software embedded in the StepOnePlus system. Primer sequences used were as follows: *GAPDH:* forward primer 5′-CTCTGCTCCTCCTGTTCGAC-3′, reverse primer 5′-ACGACCAAATCCGTTGACTC-3′; *ICAM-1:* forward primer 5′-CCTTCCTCACCGTGTACTGG-3′, reverse primer 5′-AGCGTAGGGTAAGGTTCTTGC-3′; *VCAM-1:* forward primer 5′-TGCACAGTGACTTGTGGACAT-3′, reverse primer 5′-CCACTCATCTCGATTTCTGGA-3′; *HO-1:* forward primer: 5′-GGCAGAGGGTGATAGAAGAGG-3′, reverse primer 5′-AGCTCCTGCAACTCCTCAAA-3′; *NQO-1:* forward primer 5′-CAGCTCACCGAGAGCCTAGT-3′, reverse primer 5′-GAGTGAGCCAGTACGATCAGTG-3′; *Nox1:* forward primer: 5′-TCACCCCCTTTGCTTCTATCT-3′, reverse primer: 5′-AATGCTGCATGACCAACCTT-3′; *Nox2:* forward primer: 5′-CATTCAACCTCTGCCACCAT-3′, reverse primer: 5′ -CCCCAGCCAAACCAGAAT-3′; *Nox4:* forward primer: 5′-GCTGACGTTGCATGTTTCAG-3′, reverse primer: 5′-CGGGAGGGTGGGTATCTAA-3′; *Nox5:* forward primer 5′-GGCGTCTGCAGGTACAGAGT-3′, reverse primer 5′-AGCTCATCCGGGTCAATG-3′.

### Western blotting

Human aortic endothelial cells (HAECs) (2 × 10^5^ cells/well in a 6-well plate) were stimulated with thrombin (2 U/mL) for 5 h with or without 100 nM angiotensin-(1-7) pretreatment 1 h before the thrombin stimulation. Protein expression levels in the HAECs were analyzed by western blotting. Briefly, 50 μg of each protein sample was separated by 8% sodium dodecyl sulfate polyacrylamide gel electrophoresis and transferred onto a polyvinylidene fluoride membrane by a semidry transfer method. Antibodies against Nox5 (OAEB01075, Aviva Systems Biology, San Diego, CA) and GAPDH (Santa Cruz Biotechnology, Santa Cruz, CA) were used at 1:500 and 1:1,000 dilution, respectively. Expression of the proteins of interest in the whole cell lysates was normalized to GAPDH expression.

### Nox5 gene knockdown

For Nox5 gene knockdown, HAECs were transfected with 100 nM human Nox5 siRNA (GeneDirex, Keelung, Taiwan). Scrambled negative control (NC) siRNA was included as a negative control. Briefly, HAECs were transfected using the Lipofectamine™ 2000 transfection reagent (Invitrogen, Carlsbad, CA, USA) in M-199 medium for 2 h. After transfection, the medium was changed to endothelial cell growth medium for 24 h, followed by HAEC stimulation with different experimental conditions. After treatment, expression levels of Nox5 mRNA, Nox5 protein, and HO-1/NQO-1/ICAM-1/VCAM-1 mRNA as well as ROS activity were determined.

### Inhibition of thrombin-induced phenotypic changes by N-acetylcysteine (NAC)

Cells were pretreated with the ROS scavenger N-acetylcysteine (NAC) at a dose of 10 mM for 1 h before thrombin stimulation. After thrombin treatment, the effects of NAC on different thrombin-altered phenotypes were determined.

### Statistical analysis

Statistical analyses were performed using the SPSS 12.0 statistical software (SPSS Inc., Chicago, IL, USA). All data are presented as the mean ± standard error of the mean (SEM). Pair-wise comparisons were made using a Student's *t*-test. Three or more groups were compared using one-way analysis of variance (ANOVA) with *post-hoc* Tukey tests. Significant differences were defined as *p* < 0.05.

## Results

### Angiotensin-(1-7) prevents actin cytoskeleton derangement in thrombin-simulated HAECs

**Figure 1:**
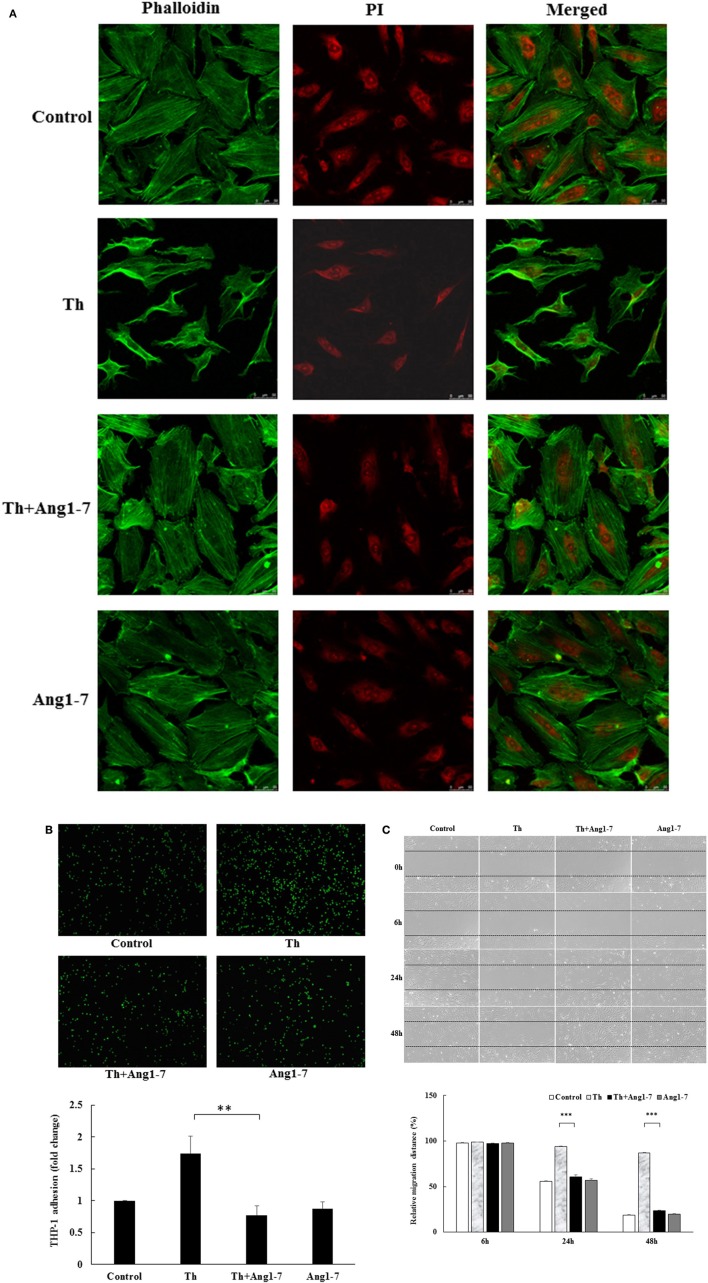
Angiotensin-(1-7) prevents thrombin-induced phenotypic changes. **(A)** HAECs were stimulated with thrombin (2 U/mL) for 5 h with or without 100 nM angiotensin-(1-7) pretreatment 1 h before the thrombin stimulation. Confocal microscopy revealed the spreading and polygonal morphology of untreated and angiotensin-(1-7)-treated HAECs. Thrombin treatment induced actin cytoskeleton derangement with variable cell retraction and increased cortical ring formation. On the other hand, thrombin-induced cytoskeleton abnormalities were prevented in HAECs pretreated with angiotensin-(1-7). Images represent results from three independent experiments. Th, thrombin. Ang1-7, angiotensin-(1-7). PI, propidium iodide. Scale bar = 50 μm. **(B)** HAECs were stimulated with thrombin (2 U/mL) for 5 h with or without 100 nM angiotensin-(1-7) pretreatment 1 h before the thrombin stimulation. Thrombin stimulation caused a significant increase in monocyte adhesion to HAECs, while angiotensin-(1-7) pretreatment caused a significant inhibition in monocyte adhesion. *n* = 6, ^**^p < 0.01 for thrombin compared with thrombin+angiotensin-(1-7) treatment. **(C)** HAECs were stimulated with thrombin (2 U/mL) for 24 and 48 h with or without 100 nM angiotensin-(1-7) pretreatment 1 h before the thrombin stimulation. Thrombin induced a significant impairment in wound-healing migration, while angiotensin-(1-7) pretreatment prevented migration inhibition at 24 and 48 h. *n* = 3. ^***^p < 0.001 for thrombin compared with thrombin+angiotensin-(1-7).

Cytoskeleton rearrangement is an early event in response to acute cellular stress. We first examined the modulatory effect of angiotensin-(1-7) on thrombin-induced changes in actin cytoskeleton using confocal microscopy. As shown in Figure 1A, untreated and angiotensin-(1-7)-treated HAECs showed spreading and exhibited polygonal morphology. In contrast, thrombin induced a significant derangement of the actin cytoskeleton, with variable cell retraction and increased cortical ring formation. Interestingly, these thrombin-induced cytoskeleton alterations were prevented in HAECs pretreated with angiotensin-(1-7), suggesting a protective effect of angiotensin-(1-7) in thrombin-induced cellular injury.

### Angiotensin-(1-7) inhibits monocyte adhesion in thrombin-stimulated HAECs

Adherence of circulating monocytes to the dysfunctional endothelium is an early event during vascular inflammation. We examined the effect of angiotensin-(1-7) on monocyte adhesion in thrombin-stimulated HAECs. As shown in Figure 1B, thrombin increased monocyte adhesion by 1.75-fold as compared to the control group. Pretreatment with angiotensin-(1-7) caused a significant decrease in monocyte adhesion. These findings support the anti-inflammatory role of angiotensin-(1-7) in thrombin-stimulated HAECs.

### Angiotensin-(1-7) repairs migration impairment in thrombin-stimulated HAECs

Upon rupture of atherosclerotic plaques, locally generated thrombin may change healing properties and promote atherosclerosis progression. As shown in Figure 1C, thrombin-stimulated HAECs displayed a significant decrease in the migratory distance, as observed by setting the relative scratch distance in different treatment groups as 100% at 0 h. The relative distance for the thrombin-treated group at 24 h was 6% of that recorded at 0 h. In contrast, the relative migratory distance was 45, 40, and 43% in the control, thrombin+angiotensin-(1-7), and angiotensin-(1-7) treatment groups, respectively. In addition, the ability of angiotensin-(1-7) to prevent thrombin-induced migration impairment was significant at 48 h, with relative migratory distances of 82, 13, 77, and 80% in the control, thrombin, thrombin+angiotensin-(1-7), and angiotensin-(1-7) treatment groups, respectively. These data suggested that angiotensin-(1-7) promoted endothelial repair by inhibiting thrombin-induced migration impairment.

### Angiotensin-(1-7) decreases ROS generation and Nox5 expression in thrombin-stimulated HAECs

**Figure 2:**
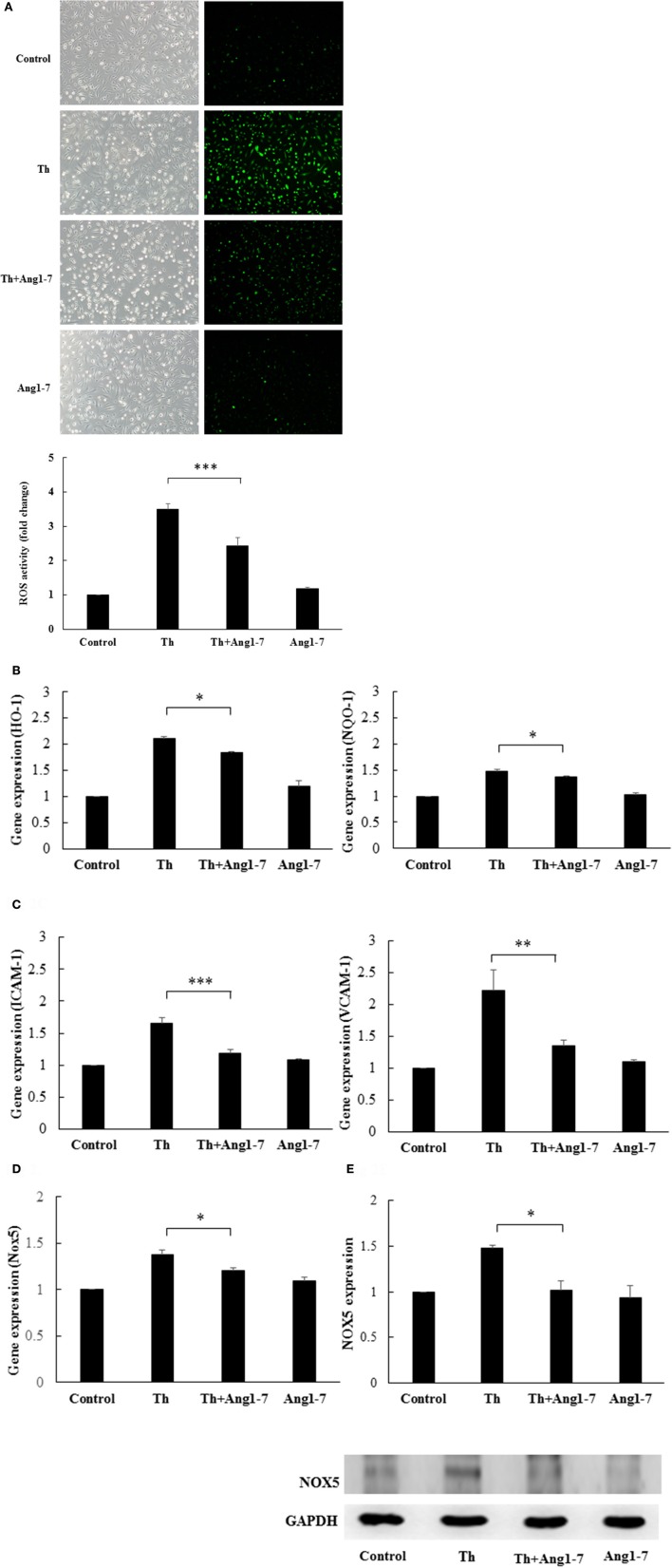
Angiotensin-(1-7) inhibits thrombin-induced ROS and Nox5 expression. HAECs were stimulated with thrombin (2 U/mL) for 5 h with or without 100 nM angiotensin-(1-7) pretreatment 1 h before the thrombin stimulation. **(A)** ROS activity assay showed that thrombin caused a significant increase in ROS production. Angiotensin-(1-7) pretreatment caused a significant decrease in ROS production. *n* = 5, ^***^p < 0.001 for thrombin compared with thrombin+angiotensin-(1-7) treatment. **(B)** Thrombin caused a significant increase in the mRNA expression of *HO-1* and *NQO-1*, while angiotensin-(1-7) pretreatment inhibited the increase in *HO-1* and *NQO-1* mRNA levels. *n* = 4, ^*^p < 0.05 for thrombin compared with thrombin+angiotensin-(1-7) treatment. **(C)** Thrombin caused a significant increase in mRNA expression of *ICAM-1* and *VCAM-1*, while angiotensin-(1-7) pretreatment decreased *ICAM-1* and *VCAM-1* mRNA expression. *n* = 4, ^***^p < 0.001 and ^**^p < 0.01 for thrombin as compared with thrombin+angiotensin-(1-7) treatment. **(D)** Thrombin caused a significant increase in the *Nox5* mRNA level, while angiotensin-(1-7) pretreatment decreased *Nox5* mRNA expression. *n* = 4, *p* < 0.05 for thrombin compared with thrombin+angiotensin-(1-7). *GAPDH* was used as the internal control for data normalization in all real-time PCR analyses. **(E)** Thrombin induced significant Nox5 protein expression, while angiotensin-(1-7) decreased the thrombin-induced Nox5 protein expression level. The blot represents results from three independent experiments. ^*^p < 0.05 for thrombin compared with thrombin+angiotensin-(1-7).

Studies have shown that NADPH-derived ROS are important signaling molecules for endothelial phenotypic modulation and that both Nox2 and Nox5 are important in the generation of thrombin-stimulated endothelial ROS (BelAiba et al., 2007). Therefore, we examined whether angiotensin-(1-7) may modulate ROS production and NAPDH oxidase expression in thrombin-stimulated HAECs. As shown in Figure 2A, ROS activity assay demonstrated that thrombin caused a 3.5-fold increase in ROS production as compared to the control group. In thrombin-stimulated HAECs, pretreatment with angiotensin-(1-7) significantly suppressed ROS production from 3.5- to 2.44-fold, as compared to the control group. The NF-E2-related factor 2 (Nrf2) antioxidant response element (ARE) signaling pathway is activated in response to oxidative stress (Nguyen et al., 2009). The heme oxygnenase-1 (*HO-1*) and NAD(P)H dehydrogenase quinone 1 (*NQO-1*) genes are important downstream targets induced by ARE activation. Along with the effect on cellular ROS levels, thrombin caused 2.11- and 1.48-fold increases in the expression of *HO-1* and *NQO-1*, respectively, as compared to the control group. In thrombin-stimulated HAECs, angiotensin-(1-7) pretreatment caused significant decreases in the gene expression of *HO-1* and *NQO-1* from 2.11- and 1.48-fold to 1.84- and 1.36-fold, respectively, as compared to the control group (Figure 2B). NADPH-derived ROS are important in mediating cellular inflammation (Dworakowski et al., 2008). As shown in Figure 2C, thrombin caused 1.66- and 2.21-fold increases in the gene expression of intercellular adhesion molecule-1 (*ICAM-1*) and vascular cell adhesion protein-1 (*VCAM-1*), respectively, as compared to the control group. In thrombin-stimulated HAECs, angiotensin-(1-7) pretreatment significantly decreased the gene expression of *ICAM-1* and VCAM-1 from 1.66- and 2.21-fold to 1.19- and 1.34-fold, respectively, as compared to the control group (Figure 2C). These findings suggested that angiotensin-(1-7) played a significant role in decreasing monocyte adhesion via *ICAM-1* and *VCAM-1* downregulation.

We then examined whether angiotensin-(1-7) could modulate the Nox5 gene expression. As shown in Figure 2D, thrombin stimulation caused a 1.39-fold increase in Nox5 gene expression as compared to control, while angiotensin-(1-7) pretreatment significantly decreased thrombin-induced Nox5 gene expression from 1.39-fold to 1.22-fold, as compared to the control group. In addition, western blot analysis showed that angiotensin-(1-7) pretreatment caused a significant decrease in thrombin-induced Nox5 protein expression (Figure 2E). As shown in Supplemental Data 1, angiotensin-(1-7) did not alter the gene expression levels of Nox1, Nox2, and Nox4 in thrombin-stimulated HAECs.

Taken together, these findings showed that angiotensin-(1-7) decreased thrombin-induced ROS production as well as ARE signaling and proinflammatory pathway activation, possibly through the downregulation of Nox5 expression.

### Thrombin-induced Nox5 is functionally intact and produces ROS in HAECs

**Figure 3:**
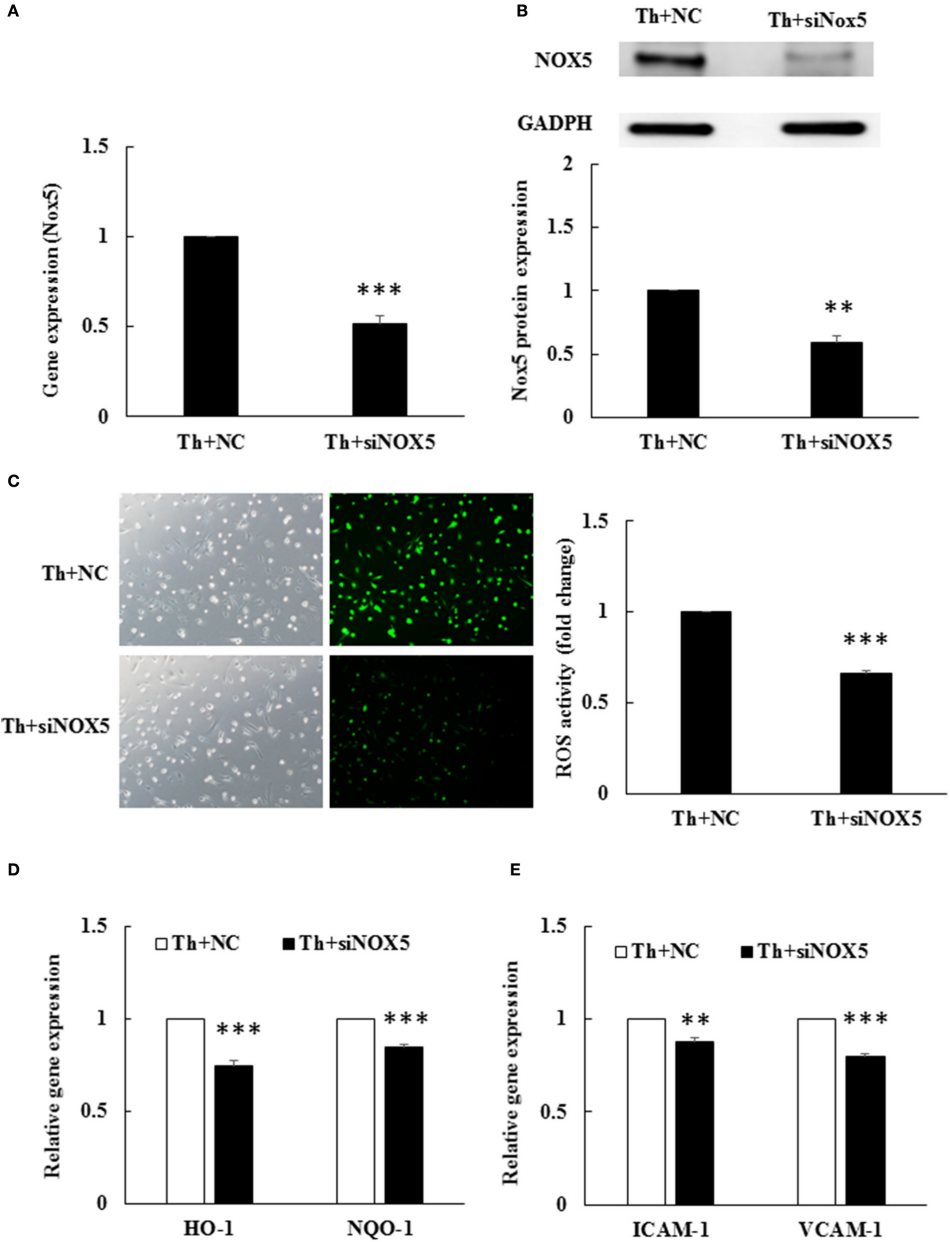
Thrombin-induced ROS production depends on Nox5. HAECs were stimulated with thrombin (2 U/mL) for 5 h with or without 100 nM angiotensin-(1-7) pretreatment 1 h before the thrombin stimulation. **(A)** Nox5 siRNA transfection caused a significant decrease in *Nox5* mRNA expression in thrombin-stimulated HAECs. NC, scrambled negative control. siNox5, Nox5 siRNA. *n* = 4, ^***^p < 0.001 for thrombin+NC compared with thrombin+siNox5. **(B)** Nox5 siRNA transfection caused a significantly decrease in Nox5 protein expression. The blot represents results from three independent experiments. ^**^p < 0.01 for thrombin+NC compared with thrombin+siNox5. **(C)** Nox5 siRNA transfection caused a significant decrease in ROS production. *n* = 5, *p* < 0.001 for thrombin+NC compared with thrombin+siNox5. **(D)** Nox5 siRNA transfection decreased *HO-1* and *NQO-1* mRNA expression. *n* = 4, ^***^p < 0.001 for thrombin+NC compared with thrombin+siNox5 **(E)** Nox5 siRNA transfection decreased *ICAM-1* and *VCAM-1* mRNA expression. *n* = 4, ^**^p < 0.01 and ^***^p < 0.001 for thrombin+NC compared with thrombin+siNox5. *GAPDH* was selected for normalization of data in all real-time PCRs.

To examine whether Nox5 expression was responsible for ROS generation in thrombin-stimulated HAECs, we conducted siRNA transfection to knockdown Nox5 expression. Nox5 siRNA significantly downregulated Nox5 gene expression as well as protein expression in thrombin-stimulated HAECs as compared with cells treated with the NC (Figures 3A,B). Furthermore, ROS production was significantly decreased in Nox5-depleted HAECs as compared to NC-treated cells (Figure 3C). The decrease in ROS generation upon Nox5 depletion was accompanied by a decrease in ARE pathway activation, as evident from the significant decreases in *HO-1* and *NQO-1* mRNA levels (Figure 3D). Moreover, both *ICAM-1* and *VCAM-1* mRNA levels were significantly downregulated upon Nox5 depletion (Figure 3E). Taken together, thrombin-induced Nox5 was functionally intact and produced ROS in HAECs. ROS, in turn, upregulated the mRNA expression of HO-1/NQO-1/ICAM-1/VCAM-1.

### Protective effect of angiotensin-(1-7) on thrombin-induced actin cytoskeleton derangement is mediated via ROS inhibition

**Figure 4:**
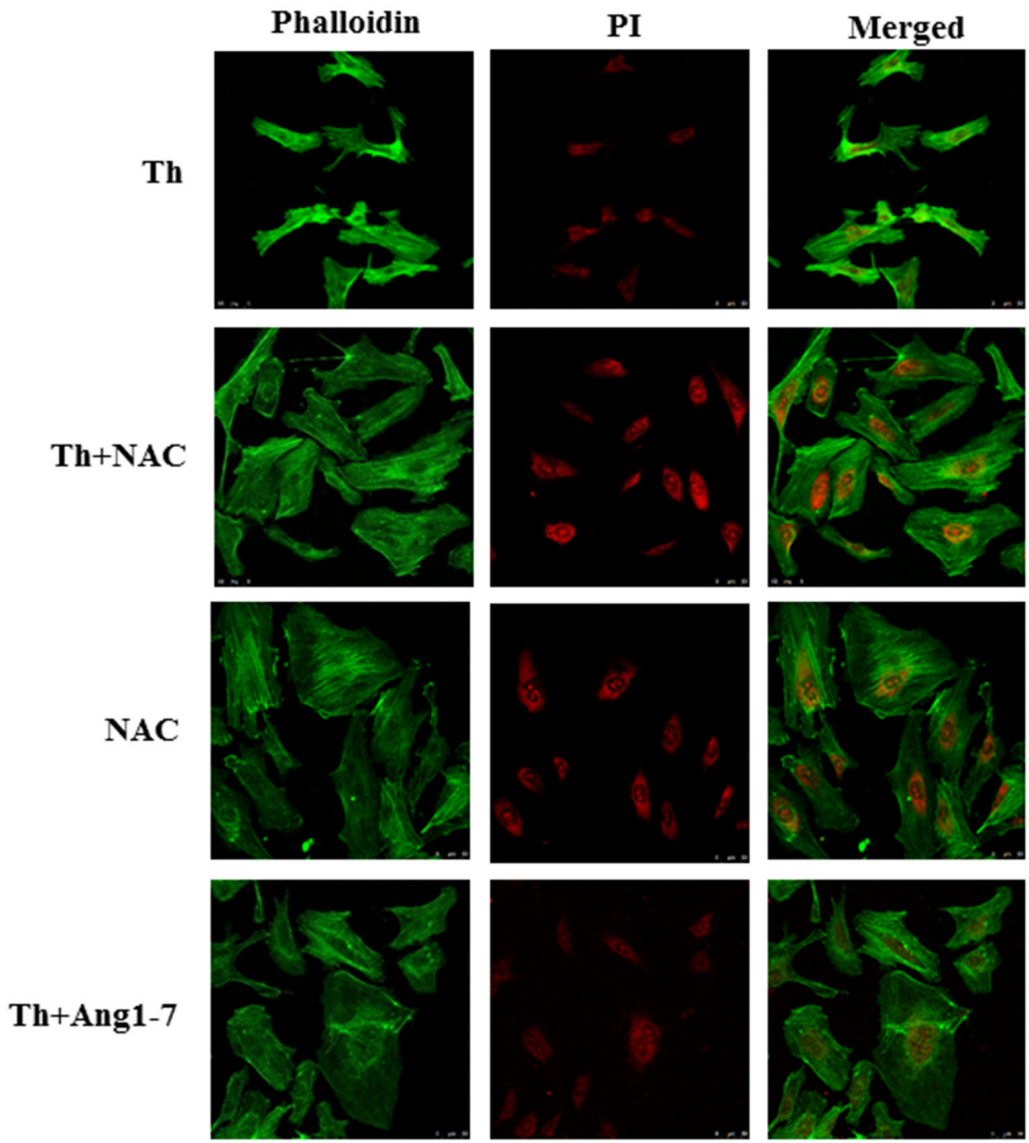
Protective effect of angiotensin-(1-7) on thrombin-induced actin cytoskeleton derangement is ROS-dependent. HAECs were stimulated with thrombin (2 U/mL) for 5 h with or without 100 nM angiotensin-(1-7) pretreatment 1 h before the thrombin stimulation. Cells were pretreated with the ROS scavenger NAC at a dose of 10 mM for 1 h before thrombin stimulation. NAC pretreatment prevented thrombin-induced actin cytoskeleton abnormalities. NAC, N-acetylcysteine. Images represent results from three independent experiments. Scale bar = 50 μm.

To examine whether the increased ROS level was responsible for actin cytoskeleton remodeling, we pretreated thrombin-treated cells with NAC. As shown in Figure 4, NAC pretreatment prevented thrombin-induced cytoskeleton abnormalities, as evident from the lack of thrombin-induced cell retraction and cortical ring formation. In addition, NAC pretreatment restored the polygonal morphology of thrombin-stimulated HAECs, similar to that observed in the thrombin+angiotensin-(1-7)-treated group. Our data supported that angiotensin-(1-7) mediated the recovery of thrombin-induced actin cytoskeleton derangement by inhibiting ROS generation.

### Protective effect of angiotensin-(1-7) on thrombin-induced monocyte adhesion is mediated via ROS inhibition

**Figure 5:**
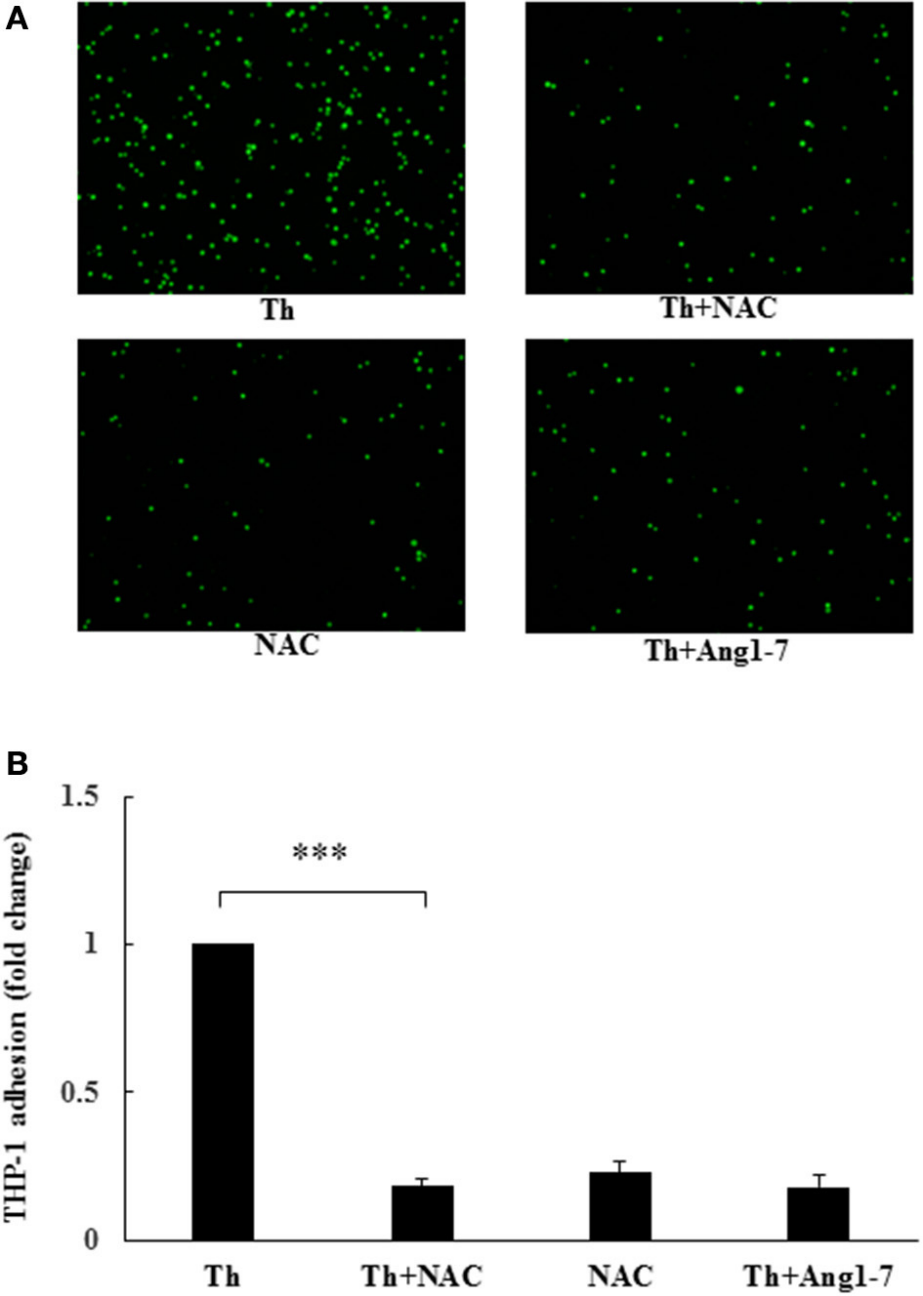
Protective effect of angiotensin-(1-7) on thrombin-induced monocyte adhesion is ROS-dependent. HAECs were stimulated with thrombin (2 U/mL) for 5 h with or without 100 nM angiotensin-(1-7) pretreatment 1 h before the thrombin stimulation. Cells were pretreated with the ROS scavenger NAC at a dose of 10 mM for 1 h before thrombin stimulation. NAC pretreatment inhibited thrombin-induced monocyte adhesion. *n* = 4; ^***^p < 0.001 for thrombin compared with thrombin+NAC.

To examine whether ROS level regulated monocyte adhesion, we pretreated thrombin-treated cells with NAC. As shown in Figure 5, NAC pretreatment in thrombin-stimulated HAECs decreased monocyte adhesion to 18% of that observed for the thrombin control group, the same percentage of that observed in the thrombin+angiotensin-(1-7)-treated group. NAC alone also decreased monocyte adhesion to 23% of that noted for the thrombin control. These results suggested that angiotensin-(1-7) mediated the inhibition of thrombin-induced monocyte adhesion by inhibiting ROS generation.

### Protective effect of angiotensin-(1-7) on thrombin-induced migration impairment is mediated via ROS inhibition

**Figure 6:**
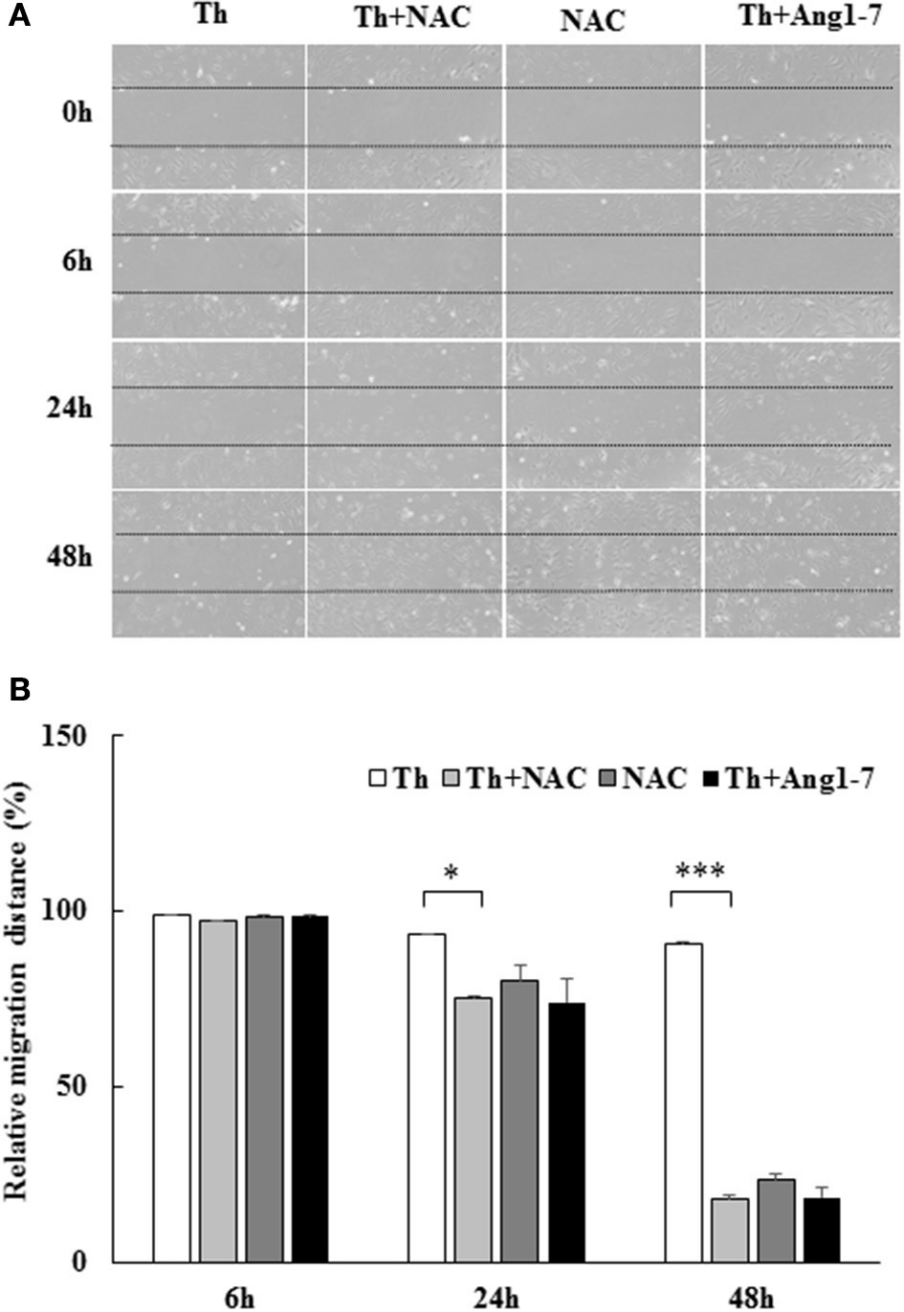
Protective effect of angiotensin-(1-7) on thrombin-induced migration impairment is ROS-dependent. HAECs were stimulated with thrombin (2 U/mL) for 24 and 48 h with or without 100 nM angiotensin-(1-7) pretreatment 1 h before the thrombin stimulation. Cells were pretreated with the ROS scavenger NAC at a dose of 10 mM for 1 h before thrombin stimulation. NAC pretreatment significantly recovered the thrombin-induced decrease in the migratory distance. *n* = 3, ^*^p < 0.05 and ^***^p < 0.001 for thrombin compared with thrombin+NAC treatment.

To examine whether the increased ROS level was involved in migration impairment, we pretreated thrombin-treated cells with NAC. As shown in Figure 6, NAC pretreatment recovered the thrombin-induced decrease in the migratory distance. At 24 h, the relative migratory distance was only 7% in thrombin group as compared to 25% in the thrombin+NAC treatment group. The migratory distance in the thrombin+NAC group was comparable to that noted for the thrombin+angiotensin-(1-7) group (26%), suggestive of the protective effect of the ROS scavenger NAC similar to that observed for angiotensin-(1-7). At 48 h, the ability of NAC pretreatment to repair thrombin-induced migratory impairment was as significant as that mediated by angiotensin-(1-7), with relative migratory distances of 9, 82, and 81% in the thrombin, thrombin+NAC, and thrombin+angiotensin-(1-7) treatment groups, respectively. Thus, the protective effect of angiotensin-(1-7) on thrombin-induced migratory impairment was mediated through the inhibition of ROS generation.

**Figure 7:**
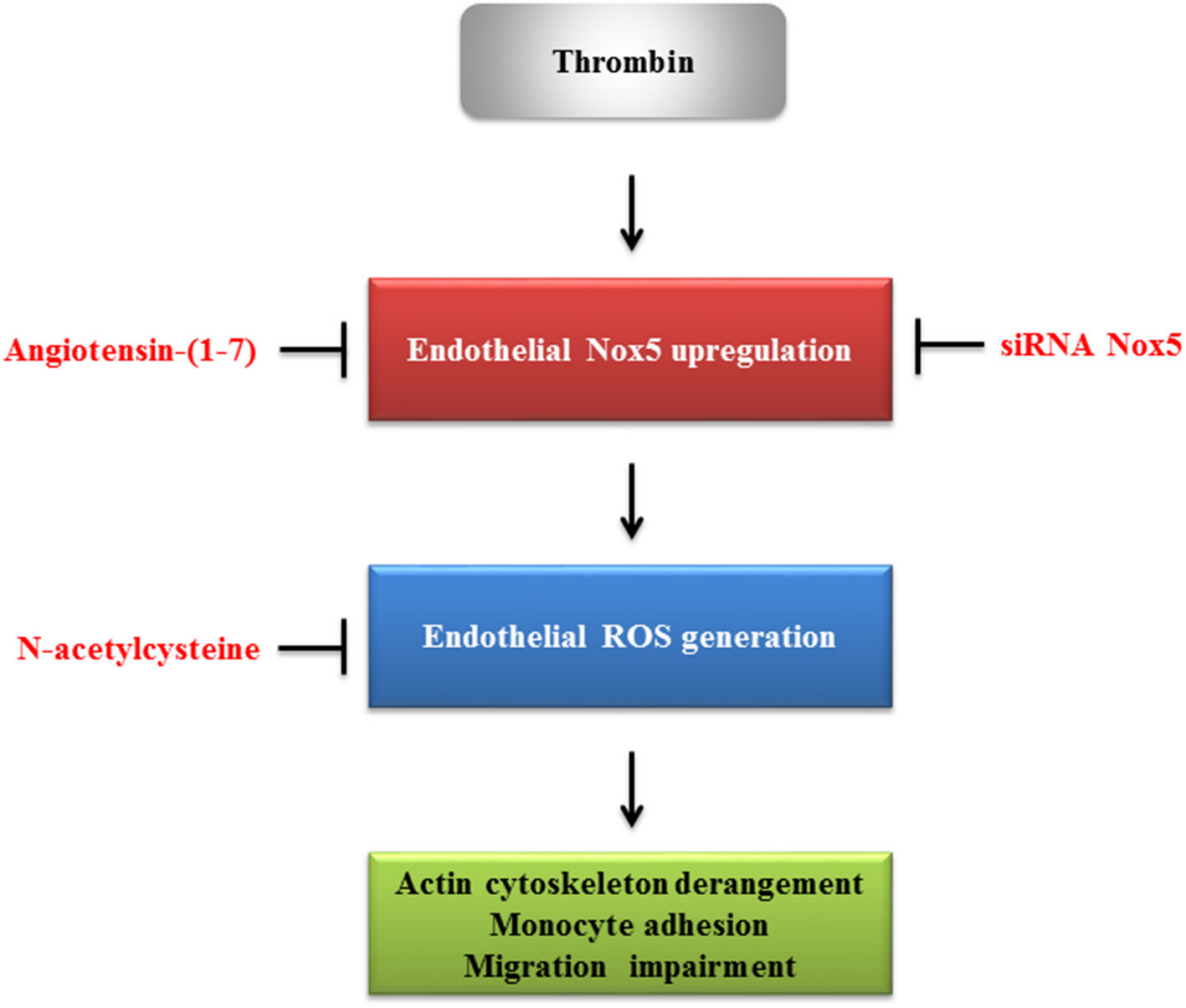
The proposed role of angiotensin-(1-7) in regulating thrombin-induced endothelial phenotypic changes.

The proposed role of angiotensin-(1-7) in regulating thrombin-induced endothelial phenotypic changes via Nox5 is shown in Figure 7.

## Discussion

The present study demonstrated the protective role of angiotensin-(1-7) against thrombin-induced phenotypic changes, including actin cytoskeleton derangement, enhanced monocyte adhesion, and migration inhibition, via downregulation of ROS production. Among multiple sources responsible for ROS production, Nox5 induction plays a significant role in ROS production in thrombin-stimulated HAECs. The acute cellular protective effects of angiotensin-(1-7) in thrombin-stimulated HAECs indicated that the downregulation of Nox5 activity may act as a potential mechanism to alleviate thrombin-induced phenotypic changes by angiotensin-(1-7).

In this study, we identified that thrombin induced significant damage to actin cytoskeleton integrity via the redistribution of F-actin into a disorganized pattern, while angiotensin-(1-7) prevented thrombin-induced actin cytoskeleton derangement. Preservation of cytoskeleton integrity by angiotensin-(1-7) was previously observed in podocytes treated with serum from preeclampsia patients (Tian et al., 2014). In this study, we showed that pretreatment of thrombin-stimulated HAECs with NAC led to a similar protective effect as angiotensin-(1-7). As angiotensin-(1-7) decreased thrombin-induced endothelial ROS activity, the protective role of angiotensin-(1-7) in thrombin-induced cytoskeleton derangement was at least partially mediated by the downregulation of ROS production.

The anti-inflammatory effect of angiotensin-(1-7) has been previously documented (Simoes and Teixeira, 2016). El-Hashim et al. reported that angiotensin-(1-7) can inhibit allergic inflammation by suppressing the phosphorylation of ERK1/2 and IκB-α in a murine model of asthma (El-Hashim et al., 2012). Al-Maghrebi et al. reported that angiotensin-(1-7) inhibited the expression of several inflammatory genes via suppression of NF-κB activity in diabetic hypertensive rats subjected to ischemic/reperfusion-induced cardiac injury (Al-Maghrebi et al., 2009). Further, Papinska et al. reported that long-term administration of angiotensin-(1-7) attenuated heart and lung dysfunction by reducing oxidative stress and inflammation in type 2 diabetic db/db mice (Papinska et al., 2016). Fraga-Silva et al. reported that angiotensin-(1-7) reduced carotid atherosclerotic plaque in ApoE knockout mice (Fraga-Silva et al., 2014). Increased cellular adhesion molecules (e.g., VCAM-1 and ICAM-1) are essential for the generation of effective endothelial inflammation (Golias et al., 2007). The adhesion of monocytes to the dysfunctional endothelium represents a hallmark for atherosclerosis initiation (Fenyo and Gafencu, 2013). Establishment of firm adhesion between endothelial cells and monocytes necessitates the binding of endothelial VCAM-1 and ICAM-1 to alpha4 integrin and beta2 integrin, respectively (Woollard and Geissmann, 2010). In endothelial cells, angiotensin II has been reported to increase VCAM-1, ICAM-1, and MCP-1 expression, while angiotensin-(1-7) attenuate the inflammatory response through the suppression of p38 mitogen-activated protein kinase (MAPK) and NF-κB signaling (Liang et al., 2015). By overexpressing ACE2 function, Zhang et al. showed that *ACE2* transfer decreased MCP-1, VCAM-1, and E-selectin expression and inhibited atherosclerotic plaque progression in ApoE-deficient mice (Zhang Y. H. et al., 2015). Thrombin signaling may convert healthy endothelium into proinflammatory phenotypes (Minami et al., 2004). In our study, angiotensin-(1-7) inhibited thrombin-induced VCAM-1 and ICAM-1 expression and monocyte adhesion, highlighting that the anti-inflammatory effect of angiotensin-(1-7) was preserved in presence of the stimulant thrombin. We observed that NAC pretreatment resulted in an inhibitory effect similar to that observed with angiotensin-(1-7) on thrombin-induced monocyte adhesion. Taken together, the above findings suggest that the protective role of angiotensin-(1-7) in thrombin-induced monocyte adhesion was mediated by inhibiting the production of ROS.

Endothelial cytoskeleton remodeling is an initial step toward cell migration (Lamalice et al., 2007). In this study, we showed that both cytoskeleton integrity and endothelial migration were impaired in thrombin-stimulated HAECs. In a previous study, thrombin has been demonstrated to stimulate the migration of porcine aortic endothelial cells (Pankonin and Teuscher, 1991). In contrast, Dimuizo et al. reported that thrombin (1–8 U/mL) inhibited scraped wound migration in a concentration-dependent manner in human iliac artery endothelial cells (DiMuzio et al., 1994). Similar to their findings, we demonstrated that 2 U/ml thrombin inhibited the wound-healing migratory distance in HAECs. These observations emphasized that the endothelial cell origin (species, organ, vessel) may influence the interpretation of findings in endothelial cell studies. As reflected in the protective effect of angiotensin-(1-7) in maintaining cytoskeleton integrity, we observed that angiotensin-(1-7) protected thrombin-induced migration impairment. Similarly to our observation, *ACE2* gene transfer was shown to promote migration in angiotensin II-stimulated endothelial cells (Zhang Y. H. et al., 2015). Furthermore, Zhao et al. reported that Mas receptor antagonist A779 significantly reduced cardiac expression of vascular endothelial growth factor and decreased angiogenesis in the infarcted myocardium of a rat model, suggesting that angiotensin-(1-7) may promote cardiac angiogenesis during the healing of myocardial infarction (Zhao et al., 2015). The increased production of thrombin at the site of atherosclerotic plaque rupture can impair endothelial healing and enhance endothelial proinflammatory phenotypes. The benefit of angiotensin-(1-7) becomes obvious at such instances, as it may speed up the healing of endothelial damage and reduce endothelial inflammation to suppress atherogenesis. In experiments using NAC, we showed that NAC pretreatment exerted a protective effect similar to that of angiotensin-(1-7) on thrombin-induced migration impairment. Taken together, the protective role of angiotensin-(1-7) in thrombin-induced phenotypic modulations, including cytoskeleton remodeling and cell adhesion and migration, is attributed to the ability of angiotensin-(1-7) to downregulate thrombin-induced ROS production.

The anti-oxidant effect of angiotensin-(1-7) is well-known (Rabelo et al., 2011). *In vitro*, Sampaio et al. showed that angiotensin-(1-7) can counteract angiotensin II signaling by attenuating the angiotensin II-induced activation of c-Src, ERK1/2, and NAPDH oxidase in HAECs (Sampaio et al., 2007a). The authors further documented that angiotensin-(1-7) can activate endothelial NO synthase and increase NO production in an AKT-dependent pathway (Sampaio et al., 2007b). Similarly, in human brain microvascular endothelial cells, Xiao et al. reported that angiotensin-(1-7) inhibited angiotensin II-induced oxidative stress via inhibition of Nox2/ROS and activation of the PI3K/NO pathway (Xiao et al., 2015). *In vivo*, Benter et al. showed that angiotensin-(1-7) prevented Nox4 gene expression and NADPH oxidase activity in the kidneys of diabetic hypertensive rats (Benter et al., 2008). Meng et al. showed that angiotensin-(1-7) attenuated bleomycin-induced lung fibrosis via downregulating the Nox4-derived ROS-mediated RhoA/Rock pathway (Meng et al., 2015). These data suggest that the attenuation of Nox2 or Nox4 activation is partially responsible for the anti-oxidant effects of angiotensin-(1-7) under different experimental conditions. Interestingly, we found that angiotensin-(1-7) predominantly inhibited Nox5 expression and ROS production in thrombin-stimulated HAECs, while the gene expression of Nox1, Nox2, Nox4 was not changed by angiotensin-(1-7) (Supplemental Data 1). BelAiba et al. reported that both Nox2 and Nox5 are equally important for mediating the generation of endothelial ROS, the proliferation phenotype, and the formation of capillary-like structure phenotype in human microvascular endothelial cells (BelAiba et al., 2007). In our data (Supplemental Data 1), thrombin did increase the gene expression level of Nox2, but Nox2 mRNA was not changed by the angiotensin-(1-7) pretreatment. In addition, siRNA Nox5 experiments suggested that Nox5 played a significant role in mediating the phenotypic changes of thrombin-stimulated HAECs. Although the gene expression of Nox2 was not changed by angiotensin-(1-7) pretreatment, we could not excluded the Nox2 activity from being changed by angiotensin-(1-7). Alternatively, the different endothelial cell types have the variable Noxs expression in response to the thrombin stimulation.

Activation of Nox proteins leads to the production of ROS, which are important signaling molecules that activate multiple downstream protein kinases [e.g., p38 MAPK, Akt/protein kinase B, c-Jun N-terminal kinase (JNK)] and redox-sensitive transcription factors [e.g., activator protein 1 (AP-1), activating transcription factor 2 (ATF-2), NF-κB, signal transducer and activator of transcription 1 (STAT1)] (Griendling et al., 2000). The expression of downstream redox-sensitive genes is important for endothelial phenotypic modulation. Of the four known endothelial Nox subtypes, Nox5 can be activated by thrombin stimulation (BelAiba et al., 2007; Wang et al., 2014). Nox5 was initially identified as a Ca^2+^-activated NADPH oxidase dominantly expressed in the testes, spleen, and lymph nodes (Banfi et al., 2001). A recent study by Guzik et al. revealed that Nox5 serves as a source of ROS in atherosclerosis. Expression of Nox5 protein and mRNA was shown to be markedly increased in coronary arteries from patients with coronary artery disease (Guzik et al., 2008). In addition, calcium-dependent and NADPH-driven ROS production (reflective of Nox5 activity) was shown to be correlated with the Nox5 mRNA level and was increased by 7-fold in these patients. In a rat model of middle cerebral artery occlusion, thrombin was shown to enhance vascular disruption (Chen et al., 2010). Angiotensin-(1-7) pretreatment in these rats significantly reduced oxidative stress levels, proinflammatory cytokines, and infarct volume, suggesting that amelioration in ROS production by angiotensin-(1-7) may offer protective effects in the clinical scenario where thrombin generation is increased (Jiang et al., 2012). In this study, we showed that angiotensin-(1-7) downregulated thrombin-induced Nox5 expression, accompanied with a decrease in ROS production. Knockdown of Nox5 expression by siRNA resulted in a remarkable decrease in ROS production, reflected by the downregulation of ARE genes *HO-1/NQO-1* and proinflammatory genes *VCAM-1/ICAM*-1. Taken together, it can be suggested that thrombin-induced Nox5 expression is involved in the production of ROS, while angiotensin-(1-7) decreases ROS through its inhibitory effect on Nox5 expression.

## Author contributions

W-YP and W-YL performed the experiments. TH, W-YL, and H-JW designed the experiments. H-JW and W-YP. wrote the manuscript. C-TP, TH, and H-JW. analyzed the experiments. C-TP, W-YL, and H-JW provided the funding. H-JW supervised the study.

### Conflict of interest statement

The authors declare that the research was conducted in the absence of any commercial or financial relationships that could be construed as a potential conflict of interest.
