# Supplementary material for: Angiotensin-(1-7) Inhibits Thrombin-Induced Endothelial Phenotypic Changes and Reactive Oxygen Species Production via NADPH Oxidase 5 Downregulation
Source: Front Physiol. 2017 Dec 8;8:994. doi: 10.3389/fphys.2017.00994 (PMC5770656; doi:10.3389/fphys.2017.00994)

**Supplemental data 1. Angiotensin-(1-7) does not inhibit thrombin-induced *Nox1*, *Nox2*, and *Nox4* mRNA expression.**

HAECs were stimulated with thrombin (2 U/mL) for 5 h with or without 100 nM angiotensin-(1-7) pretreatment for 1 h before the thrombin stimulation. Th, thrombin. Ang1-7, Angiotensin-(1-7). n = 4, NS, not significant for thrombin compared with thrombin+angiotensin-(1-7).

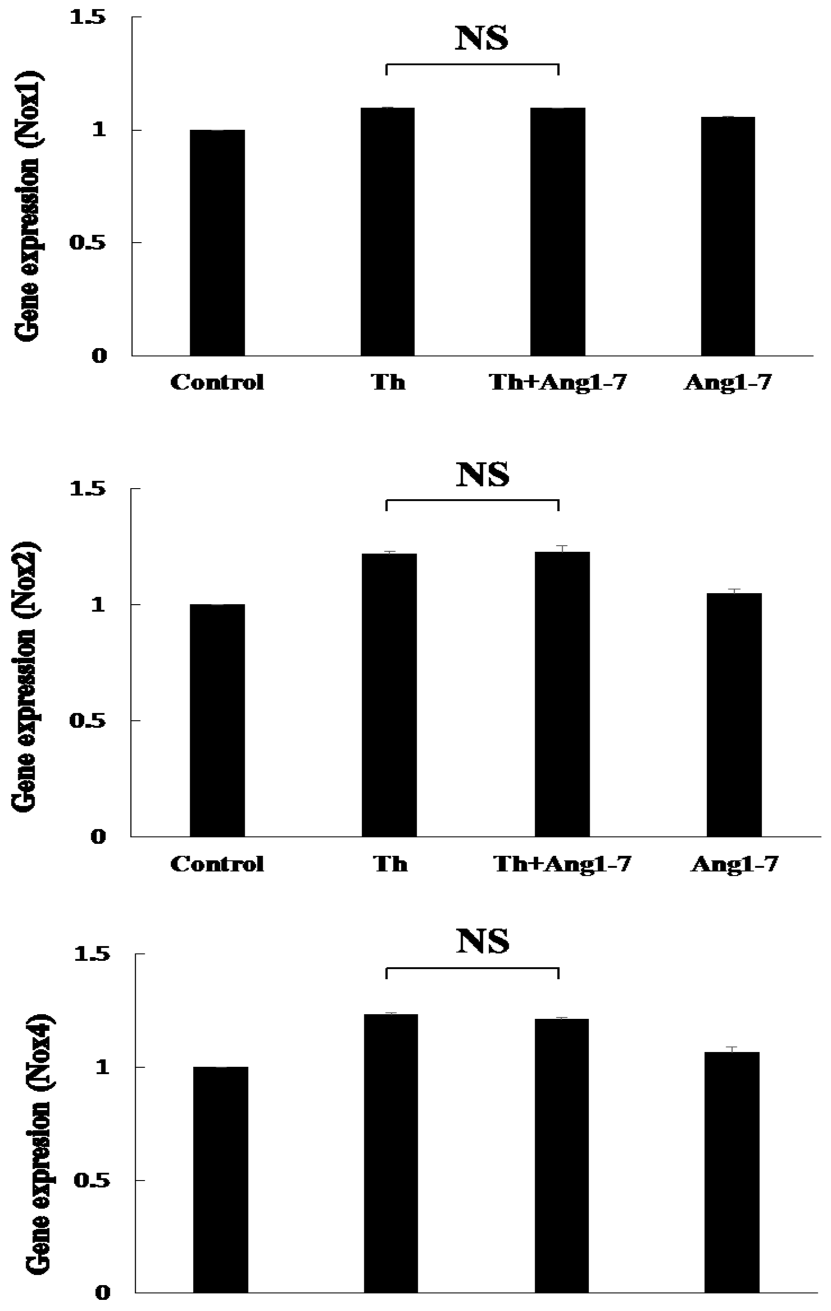

Supplement: Supplementary file 1 [file DataSheet1.PDF]
